# How the liver keeps itself in shape

**DOI:** 10.7554/eLife.85606

**Published:** 2023-02-02

**Authors:** Stephanie May, Thomas G Bird

**Affiliations:** 1 Cancer Research UK Beatson Institute Glasgow United Kingdom; 2 School of Cancer Sciences, University of Glasgow Glasgow United Kingdom; 3 MRC Centre for Inflammation Research, The Queen’s Medical Research Institute, University of Edinburgh Edinburgh United Kingdom

**Keywords:** liver, regeneration, intermittent fasting, Mouse

## Abstract

After fasting, hepatocytes proliferate to help the liver grow back to its original size.

**Related research article** Sarkar A, Jin Y, DeFelice BC, Logan CY, Yang Y, Anbarchian T, Wu P, Morri M, Neff NF, Nguyen H, Rulifson E, Fish M, Kaye AG, Martínez Jaimes AM, Nusse R. 2023. Intermittent fasting induces rapid hepatocyte proliferation to restore the hepatostat in the mouse liver. *eLife* **12**:e82311. doi: 10.7554/eLife.82311.

Are you hungry? When was the last time you consciously chose not to eat? Does fasting change the way your major organs work? Researchers performing preclinical experiments on rats and mice tend not to ask these questions, but the answers could have major implications for how the results of such experiments are interpreted.

Most lab animals are fed regularly, but this does not reflect what happens in the wild, where most rodents and other mammals (including humans) may have to undergo periods of fasting. However, the ways in which living without food affects metabolism are not fully understood. This is particularly true for the liver, which plays an important role in processing and storing energy from nutrients.

Livers are able to grow or shrink to suit the body’s requirements, and to regrow after injury (Michalopoulos and Bhushan, 2021). This ability to regnerate has fascinated scientists for centuries, as far back as the ancient Greeks: in mythology, Promethus had parts of his liver eaten every day by an eagle only for it to regrow overnight, leading to an eternity of punishment. In addition to being able to recover from damage, every day the liver produces a small number of new hepatocyte cells which are pivotal for metabolism. Now, in eLife, Abby Sarkar, Roel Nusse and colleagues at Stanford University School of Medicine and the Chan-Zuckerberg Biohub report how fasting affects the regeneration of hepatocytes in laboratory mice (Sarkar et al., 2023).

The team compared the livers of mice that had been continuously fed with mice that had been subjected to intermittent periods of living without food. Fasting on alternate days for a week altered liver metabolism, resulting in changes in fat storage and the key enzymes that produce the elements of bile, and also decreased the weight of the liver. But when the fasting mice were given continuous access to food again, their livers grew back to their original size by increasing the proliferation of their hepatocytes.

While every hepatocyte can proliferate, some are more prone to dividing and generating new cells than others, depending on where they sit within the liver (Wei et al., 2021; He et al., 2021; Chen et al., 2020; Planas-Paz et al., 2016; Lin et al., 2018). The liver is divided into small subunits called lobules that are covered in hepatocytes, and are supplied by both normal blood and blood from the intestine (Figure 1A). In continuously fed mice, the proliferation of hepatocytes happens in the mid-lobular region. However, Sarkar et al. found that proliferation happens in a different part of the liver – known as the pericentral region – in fasting mice, once they have been fed again (Figure 1B). The pericentral region has high levels of signalling through a network of proteins known as the WNT pathway, which activates another signal within hepatocytes that is crucial for development and regeneration (Russell and Monga, 2018).

**Figure 1.**
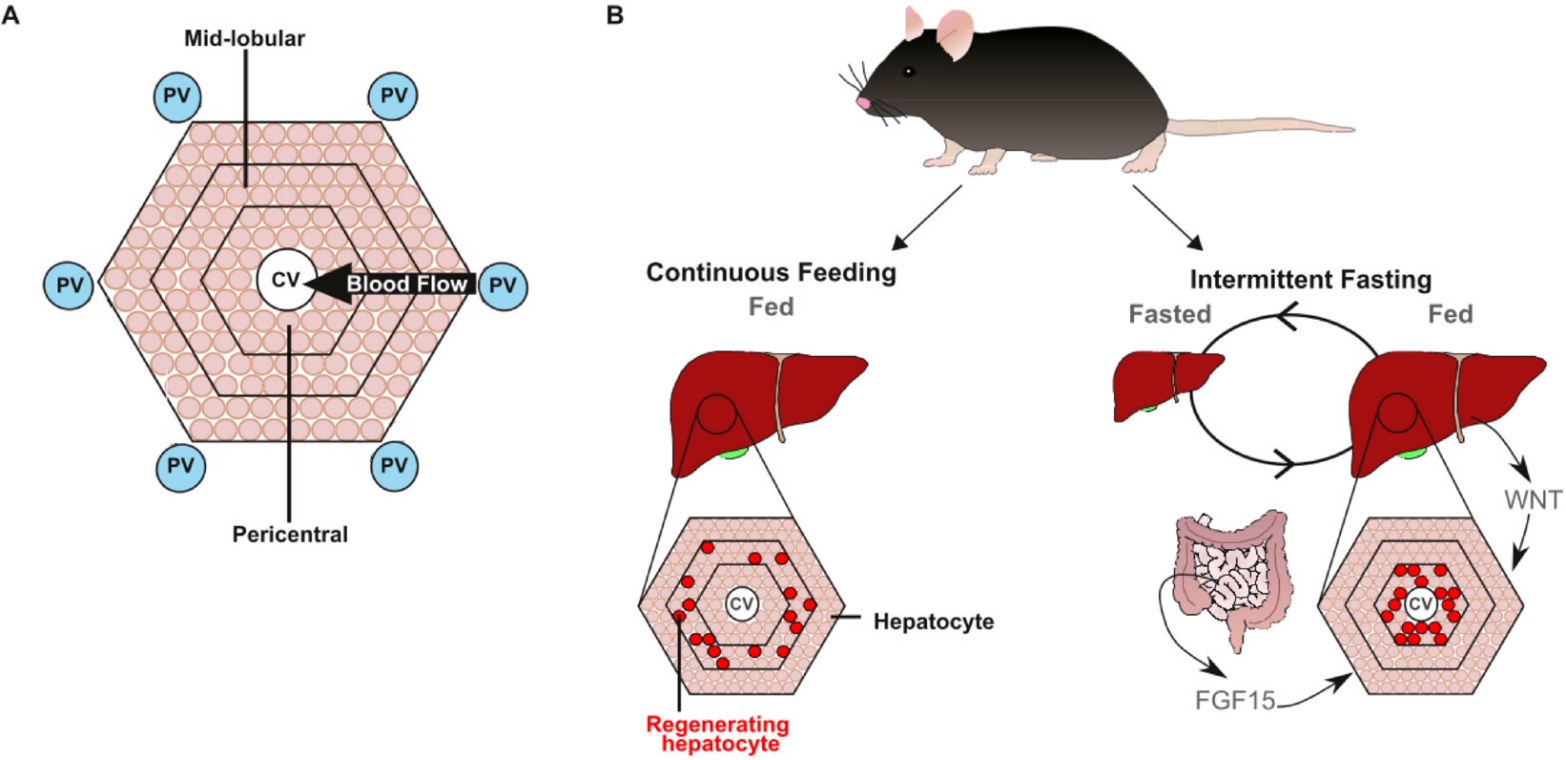
Two signals work in synergy to adjust liver size after fasting. (**A**) At the microscopic scale, the liver is divided into units called lobules which receive blood from the intestine through the portal vein (PV; blue circle). The blood passes over cells called hepatocytes (beige circles), which are responsible for most of the functions performed by the liver, and then drains out of the liver via the central vein (CV; white circle). (**B**) Some hepatocytes (highlighted in red) can also proliferate and regenerate parts of the liver. In mice that have been continuously fed (left), the hepatocytes that proliferate reside in the mid-lobular region. However, mice that have experienced intermittent periods of fasting (right) rely on a separate population of hepatocytes in the pericentral region (which is close to the central vein) to proliferate and regrow the liver once they start to eat again. These hepatocytes are activated by a signal called FGF15, which is released from the intestine when feeding restarts: the FGF15 pathway then works with the WNT pathway to stimulate proliferation.

Image credit: Stephanie May (CC BY 4.0).

Sarkar et al. then used a labelling system to mark which hepatocytes in the pericentral region responded to WNT, and tracked their progeny over time. They found a signal called FGF15, which is produced from the intestine, works together with the WNT pathway to promote regeneration when the fasted mice started eating again. By manipulating the FGF15 or WNT pathways in the liver, they showed that WNT and FGF15 synergistically drive the compensatory liver regeneration by pericentral hepatocytes after re-feeding: without FGF15 there is no growth, and without WNT the hepatocytes fail to complete cell division.

This study is the first to highlight specific changes in the regenerative machinery controlling how the liver responds to dietary fasting. It shows that pathways active within the liver can interact with signals from other parts of the body, like the intestine. The connection observed by Sarkar et al. may also be impacted by the intestine absorbing nutrients the liver then metabolizes, or bile flowing from the liver to the intestine. Whether other organs can similarly respond to fasting and/or influence regeneration elsewhere in the body remains to be seen, but the list of potential candidates is expanding (Cerletti et al., 2012).

The findings of Sarkar et al. have profound implications for how rodents and other mammals respond to fluctuations in food supply. Comparing the human body to laboratory animals that are continuously fed is likely oversimplifying biology that is vastly more complex. Fasting during development, injury or cancer may affect how multiple organs respond to a disease, both at the time and in the future. Also, how specific diets, physiological changes (e.g. pregnancy) or even differing biological sex superimpose upon this complexity remains to be explored. Nevertheless, it is clear that mammals are highly adapted to cope with periods of food unavailability, and when using animals to study health and disease, we should consider the complexity of individual dietary behaviours.
